# Pattern Recognition Receptor Ligands as an Emerging Therapeutic Agent for Latent HIV-1 Infection

**DOI:** 10.3389/fcimb.2020.00216

**Published:** 2020-05-08

**Authors:** Shokichi Takahama, Takuya Yamamoto

**Affiliations:** ^1^Laboratory of Immunosenescence, National Institutes of Biomedical Innovation, Health and Nutrition, Osaka, Japan; ^2^Laboratory of Aging and Immune Regulation, Graduate School of Pharmaceutical Sciences, Osaka University, Suita, Japan; ^3^Joint Research Center for Human Retrovirus Infection, Kumamoto University, Kumamoto, Japan

**Keywords:** TLRs, PRRs, latently HIV-1 infected cells, non-human primates, STING, immunostimulators

## Abstract

Toll-like receptors (TLRs) were first identified as molecular sensors that transduce signals from specific structural patterns derived from pathogens; their underlying molecular mechanisms of recognition and signal transduction are well-understood. To date, more than 20 pattern-recognition receptors (PRRs) have been reported in humans, some of which are membrane-bound, similar to TLRs, whereas others are cytosolic, including retinoic acid-inducible gene-I (RIG-I)-like receptors (RLRs), nucleotide-binding oligomerization domain (NOD)-like receptors (NLRs), and stimulator of interferon genes (STING). Clinically, PRR ligands have been developed as vaccine adjuvants to activate innate immunity and enhance subsequent antigen-specific immune responses. Recently, PRR ligands have been used as direct immunostimulators to enhance immune responses against infectious diseases and cancers. HIV-1 remains one of the world's most significant public health challenges. Without the elimination of HIV-1 latently infected cells, patients require lifelong combination antiretroviral therapy (cART), while research aimed at a functional cure for HIV-1 infection continues. Based on the concept of “shock and kill,” a latency-reversing agent (LRA) has been developed to reactivate latently infected cells and induce cell death. However, previous research has shown that LRAs have limited efficacy in the eradication of these reservoirs *in vivo*. Besides, PRR ligands with anti-retroviral drugs have been developed for use in HIV treatment for these years. This mini-review summarizes the current understanding of the role of PRR ligands in AIDS research, suggests directions for future research, and proposes potential clinical applications.

## Introduction

Once HIV-1 infected into CD4 T cells, it integrates into the host genome and replicates for a long time. Some of the infected cells are hidden from the immune system, and in case of immunocompromization, virions re-emerge from the latently infected cells. Even under combination antiretroviral therapy (cART), latently infected cells exist in body. Thus, curing HIV-1 infection requires elimination of HIV-1 latently infected cells, mainly located in the lymphoid organs. Several factors affect the effectiveness of viral reservoirs eradication, specifically: (1) reactivation of latently infected cells, (2) prevention of *de novo* infection by re-emerged virus produced by reactivation, (3) killing of these reactivated latently infected cells by inducing a cytopathic effect (CPE) and subsequent apoptosis and/or anti-HIV immune responses. Extensive research has been done to understand how best to use latency-reversing agents (LRAs) against HIV-1 to achieve a functional cure; these strategies have been referred to as “shock and kill” therapy (Deeks et al., 2016; Sengupta and Siliciano, 2018).

Among a variety of reagents potentially harboring LRA activity, histone-deacetylase inhibitors (HDACi) and PKC agonists have been investigated extensively and are well-documented as LRAs (Spivak and Planelles, 2018). It was initially thought that reactivation of latent HIV by LRAs would be sufficient to eliminate infected cells through CPE. However, recent data have suggested that immune effectors such as HIV-specific CTL, NK cells, or immunotoxins are likely required to recognize and eliminate exposed target cells in the so-called “flush-and-kill” strategy (Deng et al., 2015; Cartwright et al., 2016; Jones and Walker, 2016). In fact, Archin et al. have demonstrated that a single dose of vorinostat (VOR) increased the levels of cellular biomarkers of increased acetylation and simultaneously induced an increase in HIV RNA expression in resting CD4 T cells isolated from donors receiving cART (Archin et al., 2012). However, the authors did not observe any alteration in low-level viremia. This study has suggested that a single, clinically tolerable dose of VOR might be sufficient to induce the desired biological effect (histone acetylation) in PBMCs of HIV-positive, cART-treated patients. These effects were noted as temporary and were associated with increased levels of HIV RNA expression within resting CD4 T cells. Concurrently, concerns were raised about HDACi's negative impact on CTL functions (Jones et al., 2014; Clutton et al., 2016). However, a recent study by Margolis et al. has reported no measurable negative effects of HDACi on NK cell function based on *ex vivo* comprehensive immunological analysis, using PBMCs from participants treated with HDACi in two clinical studies (Garrido et al., 2019). Nevertheless, attenuated immune responses by HDACi remain subject to discussions.

Meanwhile, pattern recognition receptors (PRRs) were first identified as molecular sensors that transduce signals from specific structural patterns derived from pathogens. Their underlying molecular mechanisms of recognition and signal transduction are well-documented (Kawai and Akira, 2010, 2011; Takeuchi and Akira, 2010). To-date, over 20 PRRs have been reported; some of them are potential therapeutic targets against infectious disease or other types of disease for which there is currently no treatment. Indeed, 584 clinical trials on PRR ligands are registered at ClinicalTrials.gov, with the majority of these trials testing PRR ligands as vaccine adjuvants (Coffman et al., 2010; Reed et al., 2013; Del Giudice et al., 2018; Temizoz et al., 2018). Recently, PRR ligands as immunostimulatory drugs have received attention as potential immune therapy agents against infectious diseases and cancer, with an increasing number of trials registered at ClinicalTrials.gov. Moreover, most of the PRRs used for prospective treatment of infectious disease or cancer are agonists of TLR7, TLR8, TLR9, and STING; four clinical trials have been registered for HIV-1 treatment (Table 1). The present review summarizes the current state of knowledge regarding PRR agonists as alternative to LRAs and discusses the possible future use of these drugs as potential cure for HIV-1 infection.

**Table 1:** Selected pattern-recognition receptor agonists investigated in clinical trials for HIV, Hepatitis B/C, or cancer treatment.

**Type**	**Target PRRs**	**Drug**	**Alternative Name**	**Disease**	**Responsible Party**	**Clinical Stage**	**Identifier**
Single	TLR7	Vesatolimod	GS-9620	Hepatitis B	Gilead Sciences	Phase II	NCT01590641 NCT01590654 NCT02166047 NCT02579382
	TLR7	Vesatolimod	GS-9620	Hepatitis C	Gilead Sciences	Phase I	NCT01591668
	TLR7	Vesatolimod	GS-9620	HIV	Gilead Sciences	Phase II	NCT02858401 NCT03060447
	TLR7	RO7020531		Hepatitis B	Hoffmann-La Roche	Phase I	NCT02956850
	TLR7	TQ-A3334	AL-034	Hepatitis B	Chia Tai Tianqing Pharmaceutical Group Co., Ltd.	Phase II	NCT04180150
	TLR8	Selgantolimod	GS-9688	Hepatitis B	Gilead Sciences	Phase II	NCT03615066 NCT03491553
	TLR9	Lefitolimod	MGN1703	HIV	University of Aarhus	Phase I / II	NCT02443935
	TLR9	SD-101		Hepatitis C	Dynavax Technologies Corporation	Phase I	NCT00823862
	RIG-I	Inarigivir	GS-9992 (SB 9200)	Hepatitis B	Spring Bank Pharmaceuticals, Inc. (with Gilead Sciences)	Phase II	NCT03493698 NCT03932513 NCT04059198 NCT04023721 NCT03434353
	RIG-I	Acitretin		HIV	Ottawa Hospital Research Institute	Phase I	NCT03753867
	STING	E7766		Cancer	Eisai Inc.	Phase I	NCT04109092 NCT04144140
	STING	GSK3745417		Cancer	GlaxoSmithKline	Phase I	NCT03843359
Combination	TLR9	Lefitolimod	MGN1703	HIV	University of Aarhus	Phase I/II	NCT03837756

## Potential For Single Use of Each PRR Ligand

**Figure 1:**
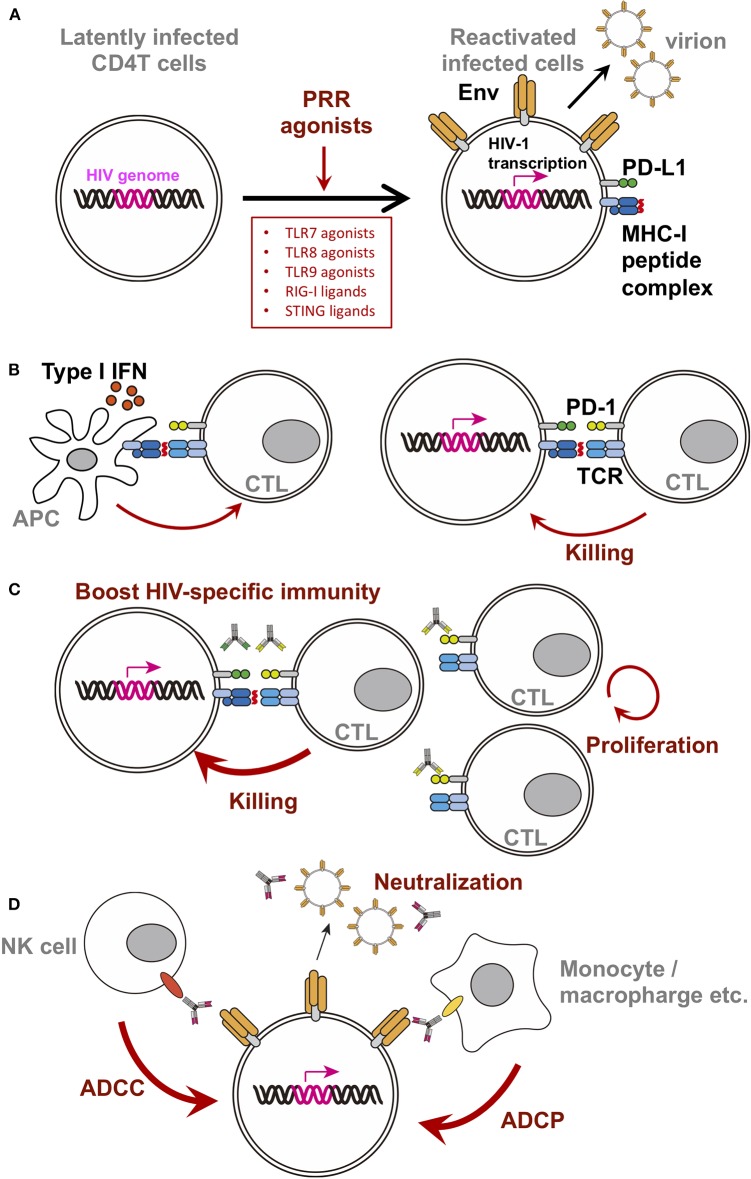
Schematic diagram of PRR agonist-induced changes in latently infected cells or surrounding players. For clarity, only selected molecules/factors are depicted **(B–D)**. Key molecules are color-coded as follows: integrated HIV-genome (magenta), surface Env (tangerine), PD-1 (yellow-green), PD-L1 (green), MHC-I/II (dark blue), peptide on MHC (red), TCR (turquoise blue), CD16 (orange), and CD32 (yellow). Each PRR ligands discussed in this mini review are written in the box. **(A)** Single treatment of PRR agonists. PRR agonist's LRA function resulted in reactivation of the latently infected cells, triggering viral gene expression, surface Env expression, and release of virions. **(B)** A simplified depiction of the consequences of the stimulation using TLR7 agonist with a therapeutic vaccine. **(C)** A simplified depiction of the consequence of using TLR7 agonist with anti-checkpoint molecules monoclonal antibodies. Blocking immune checkpoints by antibodies against PD-1 or PD-L1 revive the CTL, likely resulting in increased activity. **(D)** A simplified depiction of the consequence of using TLR7 agonist with bNab.

Among the variety of PRR ligands, ligands against TLR7/TLR8/TLR9 were studied extensively as LRA. These PRR agonist's LRA function resulted in reactivation of the latently infected cells, triggering viral gene expression, surface Env expression, and release of virions (Figure 1A). TLR7/8 senses single-stranded RNA, on the other hand, TLR9 senses unmethylated CpG-oligodeoxynucleotide-containing DNA on the endosomal membranes. These receptors are known to be expressed in pDCs and consequently produced Type I IFN upon stimulation to prevent viral infection in general. In addition, RIG-I and STING ligands are also of particular interest in the field. Thus, we will summarize the current reports of single use of these ligands.

### TLR7 Agonist

The GS-9620 is a potent and selective TLR7 agonist, originally used to treat chronic hepatitis (summarized in Table 1). Safety of oral administration of GS-9620 has been shown in phase 1/2 studies for hepatitis B (Gane et al., 2015) and hepatitis C (Lawitz et al., 2015). Jansenn et al. demonstrated the GS-9620 induced type I IFN responses assessed by ISG induction, although no significant serum IFN alpha upregulation or HBsAg level decline was observed (Janssen et al., 2018). In addition, GS-9620 has been used in the trials for HIV-1 infection (NCT02858401). For example, Tsai et al. have demonstrated that GS-9620 might induce reversal of latency in cells from HIV-infected aviremic donors on cART. Moreover, CD8 T cell, NK cell activity, and phagocytic cells activity were enhanced through Type I IFN secretion from pDC (Tsai et al., 2017). Subsequently, Lim et al. demonstrated that GS-9620 induced transient viremia and reduced viral reservoir in acutely cART-treated SIV-infected macaque model (Lim et al., 2018); 2 out of 9 treatment monkeys remained aviremic for >2 years.

### TLR8 Agonist

Recently, Meas et al. proposed the new topic of TLR8 as an LRA for HIV-1 cure strategy (Meas et al., 2020). Using human primary CD4 T cells, they demonstrated that endocytosed HIV-1 was recognized by TLR8, which in turn induces an inflammatory response that is suitable for HIV-1 replication and latency reversal. Furthermore, they also demonstrated that TLR8, but not TLR7 or TLR9, stimulation promoted differentiation to Th1/Th17 type cells, that might be contributing for HIV-1 long-term persistence in patients receiving cART (Sun et al., 2015). Thus, agonist of TLR8 would be a unique clinical target. Currently Selgantolimod (GS-9688), a selective TLR8 agonist, is intended as an immunostimulatory drug for hepatitis B (Table 1), which has been tested in animal models assessing *in vivo* safety, pharmacokinetics, pharmacodynamics, and efficacy (Daffis et al., 2017). This agent could be useful as HIV-1 treatment in the future.

### TLR9 Agonist

TLR9 agonists have been shown to reactivate latently infected cells in cART-treated patients' PBMC samples. Previous studies have shown that CpG-ODN might cause minor but significant decrease in the HIV-1 proviral reservoir (Scheller et al., 2004; Sogaard et al., 2010; Winckelmann et al., 2013), despite CpG-ODN-associated toxicity (Sogaard et al., 2010; Rynkiewicz et al., 2011; Manegold et al., 2012). In particular, a novel TLR9 agonist MGN1703 was recently developed and the effects on the reactivation of latently infected cells are well-documented (Schmidt et al., 2015; Wittig et al., 2015; Offersen et al., 2016). Søgaard et al. tested a hypothesis that MGN1703 might have dual effects (potential latency reversal and enhancement of immune function) within the “shock-and-kill” HIV-1 eradication approach. MGN1703 has been shown to induce potent antiviral responses in immune effector cells from HIV-1 infected individuals on suppressive cART *in vitro*.

Clinical studies have shown no safety concerns regarding MGN1703 (Table 1), supporting its positive effect on anti-HIV1 specific adaptive immunity (Vibholm et al., 2017, 2019). In fact, 24 weeks post-MGN1703 treatment of participants on cART, upregulation of Type I IFN and increase in the number of NK cells or proportion of activated pDC were observed. However, there was no statistically significant reduction in the number of the latently infected cells or viral load values. Furthermore, the positive effects of MGN1703 were reported within the lymph nodes and colon. In a clinical trial, Schleimann et al. demonstrated that MGN1703 enhances B cell differentiation and induction of Type I IFN responses in the lymph nodes (Schleimann et al., 2019). Similarly, Krarup et al. have demonstrated that MGN1703 induced potent type I IFN responses in colon, and that there exists an inverse correlation between the level of TLR9 and the viral DNA in colon (Krarup et al., 2018). It might be helpful to reactivate latent reservoirs that were mainly accumulated in lymphoid tissues under cART treatment (Banga et al., 2016).

### Other TLR Ligands

Data regarding reactivation of latent reservoirs by stimulation with TLR ligands is also available from *in vitro* studies. Sher et al. have demonstrated the importance of TLR2 for the induction of integrated HIV-1 expression by mycobacteria in a mouse model (Bafica et al., 2003, 2004). Subsequently, Bosque's group has demonstrated the reactivation of latently infected cells by TLR1/2 stimulation *in vitro* (Novis et al., 2013). More recently, Bosque's group has reported on the efficacy of a synthetic molecule, CL413 (or CL531/CL572), comprising TLR2 and TLR7 agonist. The compound acted as a dual TLR2/7 agonist and reactivates latency via two distinct mechanisms. As a TLR2 agonist, the compound reactivates HIV by inducing NF-kB activation in memory CD4 T cells; as a TLR7 agonist, it induces secretion of TNF-a by monocytes and pDCs, promoting viral reactivation in CD4 T cells (Macedo et al., 2018). In a cell line model, TLR3 agonist poly (I:C) efficiently reactivates HIV transcription in HIV-infected microglial cells via the IRF3 pathway without activating the NF-kB pathway (Alvarez-Carbonell et al., 2017). This effect is likely cell type-specific, as it was not observed in monocytes or T cells.

### Cytosolic PRR Ligands

Type I IFN is the best-studied downstream signaling pathway activated by PRRs. In AIDS research, the amount of Type I IFN produced by PRR ligands has been shown to correlate with the level of reactivation of latently infected cells *in vitro* (Borducchi et al., 2016; Li et al., 2016). Thus, the levels of Type I IFN production might be a marker of reactivation *in vitro*. As the stimulation of cytosolic RNA helicases (RLRs), such as retinoic acid-inducible gene-1 (RIG-I) and melanoma differentiation associated gene 5 (MDA5) enhances production of Type I IFNs, their ligands might be of interest.

In contrast to membrane-bound TLRs, RLRs detect cytosolic RNA derived from viruses (Broz and Monack, 2013; Kell and Gale, 2015). Similar to TLR agonists, the RIG-I agonist GS-9992 for Hepatitis B treatment has been conducted clinical studies (summarized in Table 1). RIG-I has been shown to detect HIV-1 RNA (Solis et al., 2011; Wang et al., 2013, 2015). Retinoic acid (RA) is a stimulator of RIG-I signaling, used as RIG-I agonist. Li et al. have reported that acitretin (an RA derivative) enhanced RIG-I signaling and increased HIV transcription, while inducing preferential apoptosis of HIV-infected cells (Li et al., 2016). Concurrently, acitretin decreased proviral DNA levels in CD4 T cells from HIV-positive participants on suppressive cART, an effect that was amplified when combined with SAHA. Acitretin also induced Type-I IFN and chemokine production. A phase-I clinical trial utilizing the oral tablet formulation of acitretin has been planned to investigate its safety and effect on the expression of several markers, including RIG-I, in CD4 T cells (NCT03753867).

Another potential candidate RLR ligand is a synthetic RLR agonist, KIN1148, which has been reported as an influenza vaccine adjuvant (Probst et al., 2017). KIN1148 induces dose-dependent expression of IRF3 and enhances the H1N1 influenza vaccine activity. However, no studies on KIN1148 as an immunostimulatory drug or LRA have been reported to-date.

Stimulator of interferon genes (STING) is another candidate for inducing strong type I IFN responses as a PRR. STING is a type of innate immune sensor for c-di-AMP (Barber, 2011, 2014, 2015; Burdette and Vance, 2013). A STING ligand, cGAMP is a promising immunomodulator, previously shown to improve tumor control in cancer studies (Corrales et al., 2016; Kinkead et al., 2018). Furthermore, several phase I trials for cancer by using STING ligands have been ongoing (selected two in Table 1). Using the PBMCs harboring of latently infected cells, previous studies have identified STING ligands as novel type of LRA, which might induce reactivation of latently infected cells and simultaneously enhancing antigen-specific CTLs (Palermo et al., 2019; Yamamoto et al., 2019). Moreover, our group has reported that another STING ligand, 3'3'-cGAMP, might induce HIV-1-specific CD8 T cells with strong effector function from naïve T cells via Type I IFN production (Kuse et al., 2019). These results suggest that STING ligands are potentially an immunostimulatory drugs for HIV.

## Combination of PRR Ligand and Other Immunotherapies

### Combination With Therapeutic Vaccines

Efficacy of TLR7 agonist for HIV infection *in vivo* was first shown in combination with a therapeutic vaccine in SIV-infected macaque model. Ad26/MVA therapeutic vaccine, a vaccine regimen primed by a recombinant adenovirus serotype 26 (Ad26), and boosted by a modified vaccinia Ankara (MVA), was administered in monkeys along with a TLR7 agonist under cART during the acute phase of infection. As a result, the set point viral load after treatment interruption alongside viral DNA in lymph nodes and PBMC were reduced, and delayed rebound was observed (Borducchi et al., 2016). Moreover, the breadth of cellular immune responses induced or boosted by this vaccine regimen inversely correlated with set point viral loads and directly correlated with time to viral rebound. The simplified depiction of the consequence of the stimulation using TLR7 agonist with a therapeutic vaccine was shown in Figure 1B (left).

### Combination With Checkpoint Inhibitors

Programmed death-1 (PD-1) blockage has been known to restore exhausted T-cell function. For clinical usage, it was first used successfully in the treatment of malignant melanoma cancers, and its effectiveness for other cancers or infectious disease has been tested. Furthermore, as a cancer immunotherapy, a combination of TLR agonists and antibodies against immune checkpoint inhibitors (e.g., PD-1, PD-L1, or CTLA4) has been proposed for clinical trials. Conceptually, immune checkpoint inhibitor compensates the barrier to attack from immune cells, while TLR agonists enhance the immune cell attack, possibly contributing to tumor size reduction.

In HIV research, upregulation of PD-1 expression in chronic HIV-1-infected patients was reported in 2006. The therapeutic vaccine boosts HIV-1-specific CTL (Figure 1B left), while CTL continues to express PD-1, which might limit CTL activity (Figure 1B right). Indeed, many groups reported the inverse correlation between the level of PD-1 expression on virus-specific CD8 T cells and VLs during chronic phase of infection (Day et al., 2006; Petrovas et al., 2006; Trautmann et al., 2006). Subsequently, PD-1 blockage enhances SIV-specific immunity *in vivo* in SIV-infected macaque model without cART (Velu et al., 2009; Dyavar Shetty et al., 2012). Furthermore, the blockage of PD-1 has been shown to enhance antiviral CTL activity and reduce the viral reservoir in SIV-infected macaque model with cART (Mylvaganam et al., 2018). These data suggested that blockage of PD-1 signaling might be a mode of treatment for HIV-1 infection (Figure 1C).

In contrast, Bekerman et al. have demonstrated the opposite result of a combination of anti-PD-1 with GS-9620 in SIV-infected chronic cART model (Bekerman et al., 2019). These authors reported no delayed rebound or changes to the size of reservoir because of treatment with PD-1 or GS-9620 used separately or concurrently, in a placebo-controlled trial. Due to the duration of cART treatment, the authors suggested PD-1 blockage might be of limited benefit. In addition to the limited benefit, the adverse effects of the checkpoint inhibitors should also be considered. In the cancer patient who has HIV-1 infection, the side effects of the PD-1 blockage appear similar to that in non-HIV patients (Scully et al., 2018). Further research on checkpoint inhibitors is required to achieve a functional cure.

### Combination With bNabs

Since the first identification of the broad neutralizing antibodies (bNabs) against HIV-1 from patient blood (Scheid et al., 2009; Walker et al., 2009; Wu et al., 2010; Zhou et al., 2010), tremendous amount of new bNabs have been developed (Kwong and Mascola, 2018; Sok and Burton, 2018; Haynes et al., 2019). In clinical studies, some of them have been shown to delay viral rebound after analytic treatment interruption (ATI) *in vivo* (Bar et al., 2016; Scheid et al., 2016). Based on these data, the Barouch group proposes a direct antiviral effect of bNabs on latently infected cells, alongside the neutralizing effect of antibodies. There might be potentially two advantages. (1) The neutralization activity against *de novo* virion produced concomitantly with the reactivation. (2) bNabs might be helpful to induce FcR-mediated killing (ADCC or ADCP) with surrounding FcR-expressing cells (Figure 1D). Using SHIV-infected macaque model treated with cART in the acute phase of infection, they demonstrated that V3 type bNab PGT121 with GS-9620 under cART delayed viral rebound following ATI (Borducchi et al., 2018). In their study, serum antibody concentration was below the detectable limit at the time of ATI, suggesting bNab's direct effect on latently infected cells. Concurrently, a combination of TLR agonists and bNabs is being studied in a Phase II clinical trial. In the study (TITAN), a combination of MGN1703 (TLR9 agonist) and 3BMC117 (anti-Env CD4 binding site bNab) will be tested with cART patients (NCT03837756).

## Importance of Animal Models for the Development of PRR Drugs

Animal studies are essential to the development of vaccines and their adjuvants. Such experiments generally involve small animals, with mouse and rat models most commonly used for efficacy and safety, respectively. Such experiments have several advantages, including relatively straightforward experimental protocols, and availability of a variety of assays alongside genetically modified mouse models required for mechanistic analyses.

In spite of these advantages, these models have several disadvantages. For example, previous studies have reported differences between mice and humans in PRR expression and innate immune responsiveness, specifically regarding innate immune response, which is relevant to research into PRR ligands as immunostimulatory drugs (Hornung et al., 2002; Pulendran, 2004; Kastenmuller et al., 2014). Moreover, TLRs 7 and 9 have been reported to show expression localized in plasmacytoid dendritic cells (pDCs) and B cells in humans; however, in mice, they are also expressed in CD8+ DC. Moreover, TLR8 expression has been reported in monocytes and conventional DC (cDC) in humans (Kadowaki et al., 2001), while functional TLR8 is not expressed in any type of mice cell, where it could be a pseudogene. These differences between mouse and human level of TLR expression might limit translation of any findings regarding PRR ligands therapeutic agents into clinical use, highlighting the need for a non-human primate model, and subsequent human trials.

Another important aspect of an animal model in HIV-1 cure studies is the timing of cART initiation. For instance, cART starting early after infection has been shown to limit the reservoir size in SIV infected macaque (Okoye et al., 2018).

Del Prete et al. have stressed the importance of cART timing. They observed no increase in viremia after serial administration of GS-9620, or changes to viral DNA associated with GS-9620 in PBMC or tissues. In their study, cART was initiated 13 days after infection and continued for 75 weeks before GS-9620 administration. Nevertheless, they observed transient upregulation of IFN-stimulated genes in blood and tissues, an increase in plasma cytokines level, changes in immune cell population activation, and phenotypes (Del Prete et al., 2019).

In chronic SIV-infected model, despite combination with PD-1 blockage, GS-9620 could not reduce the amount of viral RNA in PBMC or tissues after treatment interruption (Bekerman et al., 2019), which is in contrast to previously reported beneficial results of GS-9620 in SIV- or SHIV-infected macaque models (Borducchi et al., 2016, 2018; Lim et al., 2018). These discrepancies might be due to the timing and duration of cART, which might affect the level of set point viral loads, reservoirs size, and the likelihood of regular immune system preservation.

Altogether, data from previous studies should be carefully considered when evaluating the effect of treatment with PRR ligands based on the timing of cART initiation and its duration *in vivo*.

## Concluding Remarks

In this mini review, we discussed potential use of PRR agonists as a single agent or in combination with other LRA to eliminate latently infected cells. We focused on agonists of TLRs, STING, and RIG-I, showing that several PRR ligands might help eliminate latently infected cells (Figure 1A). However, further research is required to elucidate the underlying mechanisms of action, differences in pathway activation, crosstalk between pathways, and their metabolism *in vivo*. As most of the presented studies involved *in vitro* observations, examination of the efficacy *in vivo* is the next paramount step. In addition, an animal model treated *in vivo* with cART during a chronic phase of infection is necessary, as it would be clinically relevant to the majority of HIV-infected individuals.

In summary, progress toward a functional cure against HIV-1 in humans continues. Some of the pre-clinical studies using innate-immune activators have shown encouraging data, making it a promising candidate for a future HIV-1 cure.

## Author Contributions

All authors listed have made a substantial, direct and intellectual contribution to the work, and approved it for publication.

## Conflict of Interest

The authors declare that the research was conducted in the absence of any commercial or financial relationships that could be construed as a potential conflict of interest.
